# The integration and identity of the Romanian community in Florence in the context of life in the diaspora

**DOI:** 10.3389/fsoc.2025.1675123

**Published:** 2025-12-15

**Authors:** Denisa Ramona Chasciar, Vasile Chasciar, Claudiu Coman, Ovidiu Florin Toderici, Cristel Iotu, Adrian Otovescu, Roxana Shields

**Affiliations:** 1 Faculty of Psychology and Educational Sciences, Babeş-Bolyai University of Cluj-Napoca, Cluj-Napoca, Romania; 2 Faculty of Educational Sciences, Psychology, and Social Sciences, Aurel Vlaicu University of Arad, Arad, Romania; 3 Faculty of Social Sciences, University of Craiova, Craiova, Romania; 4 Faculty of Sociology and Communication, Transilvania University of Brasov, Braşov, Romania; 5 Faculty of Social Sciences, Doctoral School of Social Sciences and Humanities, University of Craiova, Craiova, Romania; 6 Faculty of Letter, University of Craiova, Craiova, Romania; 7 Faculty of Sociology and Communication, Transilvania University of Brasov, Brasov, Romania

**Keywords:** Romanian diaspora, migration, social integration, community identity, Florence, qualitative research, Eastern European migrants

## Abstract

This study investigates the integration and identity of the Romanian community living in Florence, analyzing how migration-related experiences shape social belonging and community cohesion within the diaspora context. The research is based on a mixed-methods design using quantitative data collected from 330 Romanian residents in Florence and qualitative data obtained through 30 interviews with Italian citizens. Quantitative findings show significant associations between migration motivations, the frequency of return to Romania, and perceived quality-of-life indicators. Qualitative results highlight themes related to cultural adaptation, social support, and intercommunity relations. The results indicate complex identity negotiations among Romanian migrants, influenced by transnational ties and interactions with the host society. The mixed-methods evidence suggests that integration is shaped by both structural and relational factors. The study contributes to understanding diaspora dynamics by offering a multi-layered perspective on integration processes within the Romanian community in Florence.

## 1 Introduction and theoretical background

Migration is one of the most complex and dynamic social processes of the twenty first century, combining economic, social and cultural transformations across borders. Within the European Union, intra-European mobility has increased steadily over the last 2 decades, particularly from Eastern to Southern and Western member states. Romania, as a major country of origin, has witnessed a significant outflow of citizens seeking employment stability and better living standards, especially to Italy and Spain (Sandu, 2010; Anghel, 2009; Blangiardo and Ortensi, 2019; Stan and Erne, 2014; Biagi et al., 2011).

According to data provided by the Italian National Institute of Statistics (ISTAT, 2023), the Romanian population remains the largest foreign community in Italy, with over one million residents legally registered. In the Tuscany region, more than 120,000 Romanian citizens live and work, many of them concentrated in Florence and its surroundings. They are employed primarily in construction, domestic care and hospitality sectors (Blangiardo and Ortensi, 2019; Giuliani et al., 2023). Despite their visible economic contribution, social integration often remains partial, with persistent challenges related to cultural adaptation and public perception (Dncic and Militaru, 2011).

Research on migrant integration emphasizes that adaptation to a host society is not limited to economic participation but also involves relational, cultural and symbolic dimensions (Ban, 2012; Uccellini, 2012; Tarabusi, 2022). In the Italian context, Blangiardo and Ortensi (2019) highlight that successful integration depends on the interplay between institutional support and community networks. Building on these perspectives, the present study examines how Romanian migrants in Florence experience integration and identity negotiation in everyday life.

Against this theoretical background, the present article explores how members of the Romanian community in Florence construct their cultural identity and perceive their social integration within the host society. The detailed research aims, objectives and methodological design are presented in the next section.

Migration is one of the most dynamic and influential contemporary social phenomena, with significant implications for the individual, the family and society as a whole (Macleod, 2012). The motivations behind migration are multiple and complex, being analyzed within numerous sociological and economic theories. An essential contribution to understanding the decision to migrate is the “push-pull” theory proposed by Lee (1966), which highlights the existence of pushing factors (such as lack of economic opportunities, poverty or political instability in the country of origin) and attraction factors (such as higher standard of living, stability and access to social services in the country of destination). These factors, in combination with the perceived obstacles and personal characteristics of the individual, profoundly influence the decision to migrate (Wickramasinghe and Wimalaratana, 2016).

Ravenstein (1885), one of the pioneers of demographic studies, formulated a series of “migration laws”, by which he emphasizes, among other things, the tendency for the majority of migrants to move short distances and to have a specific profile (young, able to work). Later, Piore (1979) introduced the concept of the “dual labor market,” suggesting that developed economies systematically create a demand for cheap labor, available especially from migrants, especially for low-skilled jobs that are unattractive to the native population.

In addition to these perspectives, contemporary theories of migration networks, such as those developed by Massey et al. (1993), emphasize the social and self-reproducible character of migration. Once an individual in a community migrates, he or she creates a bridge for others, providing information, logistical support, and the possibility of faster integration. This mechanism frequently explains the emergence of Romanian “colonies” in certain cities in Italy, such as Florence, where Romanians benefit from the support of informal networks to find a job, a home or to adapt to the local culture. Ban (2012) expands on this idea, discussing economic transnationalism, through informal recruitment networks that facilitate both mobility and temporary return or dual residency.

However, the process of social integration is not linear or completely guaranteed. According to the definition proposed by Zamfir and Zamfir (2003), integration implies the active and equal participation of migrants in all spheres of social life–economic, educational, civic, and cultural. This is not just about physical presence in a space, but involves access to resources, recognition, and a sense of belonging. Integration is a multidimensional process, with different rhythms and depths, influenced by factors such as professional status, educational level, knowledge of the language of the host country and the attitude of the majority community toward migrants. Studies by Blangiardo and Ortensi (2019) and (Panizzon and Van Riemsdijk 2019) support the importance of a multi-level institutional framework in facilitating this process, highlighting the role of local and regional authorities in defining integration policies.

In the case of Romanians in Italy, integration is often partial, marked by economic successes, but also by relational and cultural difficulties. Dncic and Militaru (2011) draw attention to the discrepancy between participation in the labor market and integration into the social and civic life of the host country. Uccellini (2012) completes by discussing the insider/outsider status of Romanian migrants and revealing that belonging depends on access to resources and social recognition. Tarabusi (2022) emphasizes how community identity is constructed locally, influencing the restoration of civic belonging.

In parallel with integration, the preservation of cultural identity plays a crucial role in the diaspora experience. Migration does not mean abandoning national identity, but reconfiguring it in a new context. Romanians in the diaspora often experience a process of “dual belonging”, in which they try to adapt to the cultural norms of the host country, without completely renouncing the values and traditions of their country of origin (Andrén and Roman, 2016). Vertovec (2001) discusses the identity of the diaspora as dynamic, stratified and constantly negotiated according to the social, political and economic context, and Parati (2013) analyzes the way in which Romanians “speak back” to stereotypes, affirming their identity through cultural discourse.

In support of the preservation of identity, community structures—churches, Romanian associations, informal schools or cultural centers—frequently intervene. They function as “identity islands” and contribute to the internal cohesion of the community. Giuliani et al. (2023) and Poenaru (2020) also highlight the role of transnational networks and migrant entrepreneurship in strengthening identity and economic integration. Vlase and Voicu (2014) examine how ethnic structures use agency strategies to navigate between social constraints and individual choices.

In conclusion, the literature provides a solid framework for understanding migration and integration, highlighting the complexity of these phenomena. The combination of classical theories (Lee, Ravenstein, Piore, Massey) with contemporary perspectives on governance, social representations, and the role of identity allows for a coherent analysis of the way in which Romanians in Florence live the experience of migration, adapt and negotiate their identity in an intercultural space (Bonifazi et al., 2021).

## 2 Research methodology

### 2.1 Purpose and objectives

The purpose of this article is to analyze how the members of the Romanian community in the city of Florence, Italy, experience the process of social integration and negotiate their cultural identity within the host society, from the perspective of their own perceptions and the perceptions of Italian citizens.

In line with the stated purpose, the research proposes the following specific objectives:

**O1:** Identify the main reasons that determine Romanians to migrate to Italy and to choose Florence as a destination, in connection with family and economic motivations;
**O2:** Analyze the role of social and family networks in the migration process and in migrants' decisions to settle in Florence;
**O3:** Examine transnational mobility patterns among Romanians in Florence, including the frequency, purpose and social factors influencing return visits to Romania;
**O4:** Assess the perceived quality of life, satisfaction, and degree of social integration of Romanians living in Florence, as reflected in their interaction with Italian citizens;

### 2.2 Hypotheses

**H1**. The main reasons that determined the migration of Romanians to Florence are of an economic and family nature.
**H2**. The longer the period of stay in Italy, the higher the level of satisfaction with life.
**H3**. The Romanian community is perceived more negatively by Italian citizens than by its members.

To test this hypothesis (H3), both Romanian and Italian participants answered the same 5-point Likert-type item: “Overall, how positive is your perception of the Romanian community in Florence?” (1 = very negative, 5 = very positive). Using the same wording and identical response scale allows a direct and methodologically valid comparison between the two groups.

Each hypothesis is directly related to the specific objectives formulated above, ensuring internal consistency between the theoretical framework, research questions and data analysis.

### 2.3 Sample

The research was carried out on two categories of participants. The first category included a sample of 330 Romanian citizens settled in the Florence region, selected on the basis of the following criteria: Romanian citizenship, current residence in Italy and minimum age of 18 years. The participants completed an online sociological questionnaire, distributed through social networks, community organizations and direct contacts. The sample is heterogeneous in terms of age, gender, level of education, length of stay in Italy, and employment status.

The second category consisted of 30 Italian citizens living in the same region, selected by the availability method. The Italian participants were recruited through the availability (convenience) sampling method. This means that respondents were selected based on their accessibility and willingness to participate. Potential participants were approached in public spaces such as parks, cafés, local community centers, and workplaces, and were invited to take part in the study if they met the inclusion criteria (Italian citizenship, age over 18, residence in the Florence area). This non-probabilistic sampling technique is commonly used in qualitative research to obtain rich and contextually relevant information. They participated in semi-structured interviews, meant to capture their perceptions of the Romanian community in the area. The inclusion of this qualitative dimension allows the triangulation of data and a better contextualization of the results obtained.

### 2.4 Research methods and tools

The study used a mixed methodological approach, which combined quantitative and qualitative research techniques, in order to obtain a complex and balanced perspective on the analyzed phenomenon. For the comparison of dependent nominal proportions, pairwise McNemar tests with Bonferroni correction were applied (Creswell, 2018).

The quantitative component was represented by the application of a structured sociological questionnaire to a sample of 330 Romanians living in the Florence region. The questionnaire included closed and semi-closed questions and targeted dimensions relevant to the research objectives, such as: migration motivation, persons involved in the migration process, frequency of return to Romania, elements of quality of life (satisfaction, professional stability, access to services, social relations), as well as aspects related to identity attachment.

The qualitative component consisted of 30 semi-structured interviews conducted with Italian citizens from the same area. This stage aimed to explore the perceptions of the local population regarding the Romanian community, bringing depth and interpretative context to the data obtained through the questionnaire. The interviews allowed for a nuanced understanding of intercultural interactions and how cultural differences are perceived.

The research question that guided the quantitative part of the research is: *How do Romanians in Florence perceive the integration process and to what extent do they maintain their cultural identity in the context of life in the diaspora?*

This question was the starting point in the formulation of hypotheses and research tools, ensuring coherence and relevance to the entire methodological approach.

## 3 Results

### 3.1 Quantitative research results

The following are the results of the research obtained from the application of the questionnaire among the members of the Romanian community in the Florence region. The data analysis is carried out according to the objectives formulated above, having as a starting point the general objective of the study: to investigate the way in which the Romanians settled in this area define themselves. Within this framework, a series of specific objectives were established, which guided the process of data collection and interpretation, as well as relevant dimensions for the deep understanding of the life experience of the members of the Romanian community in the Italian diaspora.

**O1: Identify the main reasons that determine Romanians to migrate to Italy and to choose Florence as a destination, in connection with family and economic motivations**.

According to the results of the research, the main reason why Romanians emigrated to Firenze is to obtain a better paid job than in Romania (45.2%). Also, a significant percentage of them (19.4%) said that they migrated to Firenze to have a higher standard of living, and 16.1% left Romania to be able to help their family who remained at home. Moreover, 9.7% of respondents said that they also migrated to reunite their family (Table 1).

**Table 1:** The reasons why Romanians emigrated to Florence.

**Valid**	**Response option**	**Frequency**	**Percent**
	To get a better paying job	149	45.2
	To have a higher standard of living	64	19.4
	For family reunification	32	9.7
	To help my family who remained in Romania	53	16.1
	Another	32	9.7
	Total	330	100.0

The research also aimed to identify the reasons why Romanians chose to settle in Florence. The results reveal that most of the respondents were attracted by the working conditions (48.4%), and for many of them (32.3%) it mattered that their friends already lived in this area. Moreover, 6.5% of them settled in Firenze because their relatives lived in the area (Table 2). It can thus be said that the migration of Romanians to Florence was also determined by the social networks of Romanians.

**Table 2:** The reasons why Romanians chose to settle in Florence.

**Valid**	**Response option**	**Frequency**	**Percent**
	Working conditions	160	48.4
	My friends live in this area	106	32.3
	Other relatives live in this area	21	6.5
	Due to the climate	11	3.2
	It's a quiet area	11	3.2
	It is an area with many entertainment opportunities	11	3.2
	Others	10	3.2
	Total	330	100.0

**O2: Analyze the role of social and family networks in the migration process and in migrants' decisions to settle in Florence**.

Taking into account the people with whom Romanians went to Florence, the results of the research reveal that most of them left the country alone (77.4%), many of them left with their husband or wife, 19.4%, and 3.2% of them traveled with friends (Table 3). It can be seen that most Romanians chose to leave the country alone, while the number of those who left accompanied is quite small.

**Table 3:** The people with whom Romanians migrated to Firenze.

**Valid**	**Response option**	**Frequency**	**Percent**
	Alone	255	77.4
	With parents or children	64	19.4
	With friends	11	3.2
	Total	330	100.0

Most respondents migrated alone, while smaller groups reported moving with a spouse, partner, or family members. These results confirm the primarily individual character of the first migration waves, followed by subsequent family reunification.

**O3: Examine transnational mobility patterns among Romanians in Florence, including the frequency, purpose and social factors influencing return visits to Romania**

Regarding the frequency with which Romanians from Florence return to the country, it is noted that most of them decided to give neutral answers (39.7%), saying that they do not return to the country rarely or often. In other words, most of the respondents remained impartial regarding the frequency of returning to Romania. However, a significant percentage of them (22.6%) return home quite often, 19.4% of them return quite rarely, and 12.9% of them return extremely rarely (Table 4).

**Table 4:** The frequency with which Romanians from Firenze return to Romania.

**Valid**	**Response option**	**Frequency**	**Percent**
	Extremely rare	43	12.9
	Rarely	21	6.5
	Quite rare	64	19.4
	Neither rarely nor often	128	38.7
	Quite often	74	22.6
	Total	330	100.0

A Chi-square test of independence was conducted to examine whether the frequency with which Romanians in Florence return to Romania differs by marital status. To meet the requirements of the test, the ordinal frequency scale was collapsed into two categories (rare return and frequent return). The analysis revealed a statistically significant association between marital status and return frequency, χ^2^(1) = 7.77, *p* < 0.01, indicating that married respondents tend to return to Romania more often than unmarried ones. The results of this analysis are presented in Table 5.

**Table 5:** Chi-square test for the association between marital status and frequency of return to Romania.

**Frequency of return**	**Married (*n* = 76)**	**Unmarried (*n* = 24)**	**Total**
Rare return (1–3)	27	16	43
Frequent return (4–5)	49	8	57
Total	76	24	100

According to the results of the research, most Romanians living in Florence return to Romania to spend their holidays (54.8%). Many of them return to visit relatives (25.8%), and to spend the Easter or Christmas holidays (12.9%) (Table 6). In this regard, it can be said that the Romanians from Florence return to Romania for relatively short periods.

**Table 6:** The main reasons why Romanians in Florence are returning home.

**Valid**	**Response option**	**Frequency**	**Percent**
	Spending your vacation	181	54.8
	Spending the winter/Easter holidays	43	12.9
	Visiting your relatives	85	25.8
	Alt motif	21	6.5
	Total	330	100.0

**O4: Assess the perceived quality of life, satisfaction, and degree of social integration of Romanians living in Florence, as reflected in their interaction with Italian citizens**.

### 3.2 Life satisfaction

**Figure 1:**
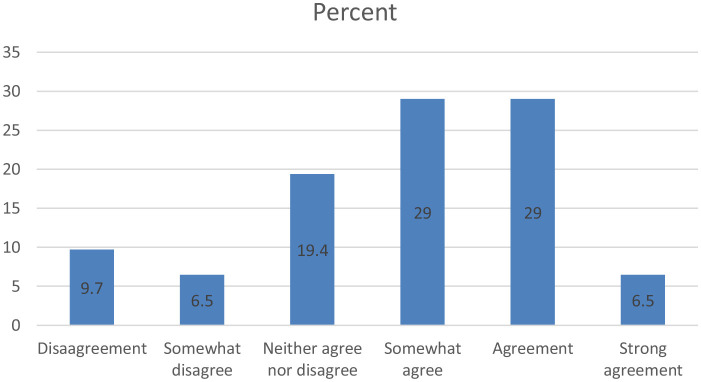
The opinion of the Romanians in Florence about the statement: In general, my life is close to my ideal.

Given life satisfaction, most respondents somewhat agree (29%) and agree (29%) with the statement that their life is close to the ideal they have. A significant percentage of respondents gave neutral answers (19.4%), and 9.7% of them strongly disagreed with this statement (Figure 1). It can thus be said that in general, the majority of Romanians in Florence consider that their life is close to the ideal they have created. Since life satisfaction was measured through a single-item statement, the result should be interpreted with caution regarding reliability. Nevertheless, the finding is consistent with previous studies showing that life satisfaction among migrants increases with time spent in the host country (Kogan and Shen, 2019).

### 3.3 Access to medical and social services

**Figure 2:**
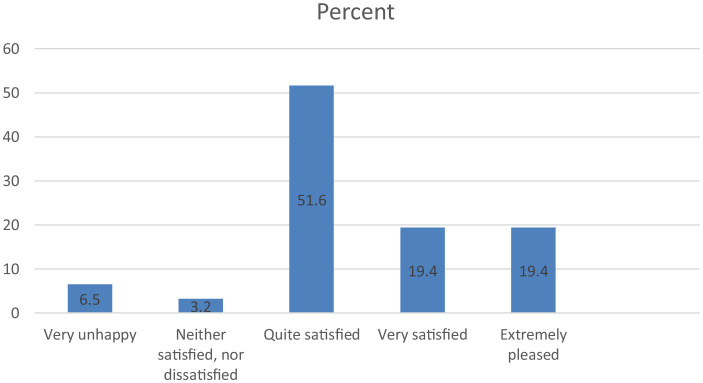
Romanians' satisfaction in Florence with medical services.

Regarding medical services, most respondents say they are quite satisfied with the medical services they have access to in Florence (51.6%). Also, 19.4% of them say they are very satisfied and even extremely satisfied. In this regard, it can be said that Romanians consider that they have quality medical services in Florence (Figure 2).

### 3.4 Financial situation and job stability

During the research, we were also interested in identifying the level of satisfaction of Romanians in Florence with the salary they receive and with the stability of their job. In this regard, the respondents are quite satisfied with the financial remuneration they receive (48.4%), then 19.4% of them have neutral opinions, and 16.1% of them are even extremely satisfied (Table 7). Thus, it can be said that Romanians in Florence consider that the jobs they have are well paid, their level of satisfaction with the salary being high.

**Table 7:** Romanians' satisfaction in Florence with the salary they receive.

**Valid**	**Response option**	**Frequency**	**Percent**
	Very dissatisfied	11	3.2
	Quite unhappy	11	3.2
	Neither satisfied nor dissatisfied	64	19.4
	Quite satisfied	160	48.4
	Very satisfied	32	9.7
	Extremely satisfied	52	16.1
	Total	330	100.0

As for job stability, most Romanians in Florence are quite satisfied (51.6%). At the same time, 12.9% of them are very satisfied, and 6.5% of them are extremely satisfied. It can be seen that the answers with positive valences registered higher percentages than the answers with negative valences, and we can deduce that Romanians in Florence managed to obtain stable jobs and that they are generally satisfied with the stability they have from a professional point of view (Table 8).

**Table 8:** Romanians' satisfaction in Florence with job stability.

**Valid**	**Response option**	**Frequency**	**Percent**
	Extremely dissatisfied	11	3.2
	Very dissatisfied	32	9.7
	Quite unhappy	11	3.2
	Neither satisfied nor dissatisfied	43	12.9
	Quite satisfied	170	51.6
	Very satisfied	43	12.9
	Extremely satisfied	20	6.5
	Total	330	100.0

### 3.5 Social integration and sense of belonging

In the context of the social environment, we were interested to find out if Romanians consider that they are treated with more respect in Florence compared to Romania and if they feel that they are treated differently because of their nationality.

**Figure 3:**
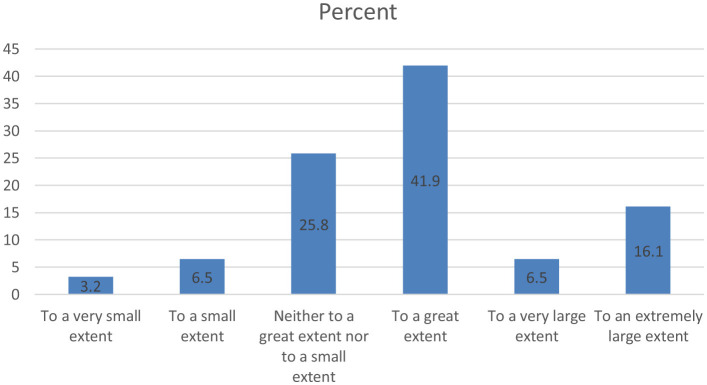
Romanians' opinion in Florence about the respect received in Italy compared to Romania.

In this regard, most respondents (41.9%) said that they feel more respected in Italy than in Romania to a large extent. Also, 25.8% of them had neutral opinions, and if we analyze the answers with positive valences (to a large extent, to a very large extent, to an extremely large extent), compared to the answers with negative valences (to a small extent, to a very small extent, to an extremely small extent), it can be seen that the number of Romanians in Florence who gave positive answers (64.5%) is significantly higher than the number of those who gave negative answers (9.7%) (Figure 3). It can thus be said that Romanians in Florence consider that they are treated with more respect than in Romania.

Given the feeling that they were treated differently in Italy because of their nationality, the majority of respondents said they did not feel they were treated differently (58.1%). However, a significant percentage of them stated that they felt they had received different treatment (32.3%) (Table 9).

**Table 9:** Romanians' opinion in Florence about the way they were treated in Italy because of their nationality.

**Valid**	**Response option**	**Frequency**	**Percent**
	Yes	106	32.3
	No	192	58.1
	I don't know/don't answer	32	9.7
	Total	330	100.0

### 3.6 Leisure and recreational activities

**Figure 4:**
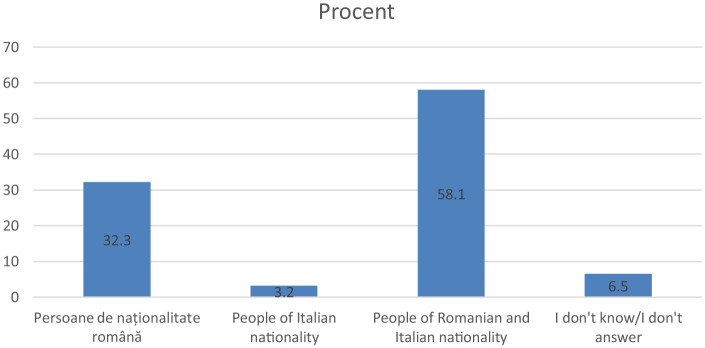
Who do Romanians in Florence prefer to spend their free time with.

In terms of leisure, Romanians in Florence prefer to spend time with both Romanian and Italian nationals (58.1%). However, there is also a high percentage of respondents who stated that in their free time they prefer to carry out activities with people of Romanian nationality (Figure 4).

Taking into account the activities that Romanians in Florence prefer to carry out in their free time, it can be seen that most respondents said they like to walk outdoors (41.9%), and many of them (38.7%), said they like to meet friends and colleagues. At the same time, to a lesser extent, there are also respondents who mentioned that they prefer to read in their free time (9.7%), play sports (6.5%) or watch movies/series (3.2%) (Table 10).

**Table 10:** Activities that Romanians in Florence prefer to do in their free time.

**Valid**	**Activity type**	**Frequency**	**Percent**
	Read	32	9.7
	You walk in the open air	138	41.9
	You meet up with friends or colleagues	128	38.7
	Carry out sports activities	21	6.5
	You watch movies/series	11	3.2
	Total	330	100.0

### 3.7 Qualitative research results

In addition to the quantitative analysis carried out by applying questionnaires, the study also included a qualitative component, meant to deepen the data obtained and to provide a contextualized understanding of social perceptions. Thus, semi-structured interviews were conducted with Italian citizens from the Florence region, in order to capture attitudes toward the Romanian community and to highlight the way in which this presence is perceived in the host society.

The qualitative component consisted of 30 semi-structured interviews conducted with Italian citizens living in Florence. The interview guide included five open-ended questions addressing the following themes:

(1) General perceptions of the Romanian community;
(2) work-related experiences and professional interactions;
(3) Participation in social and cultural activities;
(4) Media representations of Romanians;
(5) Examples of intercultural communication in everyday life.

The qualitative component consisted of 30 semi-structured interviews conducted with Italian citizens living in Florence and the surrounding area. The interview guide included five open-ended questions exploring: (1) general perceptions of the Romanian community; (2) work-related experiences and interactions; (3) participation in local cultural and social life; (4) media representations of Romanians; and (5) everyday intercultural communication. Each interview lasted between 20 and 40 mins and was audio-recorded with the participants' consent.

The themes summarized above were further detailed in the qualitative analysis that follows. These thematic categories are summarized in Table 11, which outlines the main dimensions identified in the qualitative analysis.

**Table 11:** Main themes identified from the qualitative analysis.

**Theme**	**Description**	**Illustrative quote**
Economic contribution	Italians emphasized Romanians' hard work, especially in construction and domestic care sectors.	“Romanians are among the most reliable workers in my company.” (M., 41)
Social participation	Most respondents noted low involvement of Romanians in local cultural or community events.	“They come to work and then go home, they don't mix much.” (C., 52)
Interpersonal relations	Direct contact led to more positive attitudes and friendships.	“My neighbor is Romanian — we help each other all the time.” (A., 37)
Stereotypes and media influence	Negative perceptions often came from media coverage, not personal experience.	“The news exaggerates; the Romanians I know are polite and respectful.” (L., 45)
Cultural identity	Italians perceived that Romanians preserve their traditions while adapting to Italian norms.	“They keep their holidays, but they also celebrate with us.” (S., 29)

The interviewees' answers reveal a wide range of perspectives. On the one hand, a significant part of those interviewed appreciate the contribution of Romanians to the local labor market, especially in areas such as construction, elderly care, and cleaning. They are considered hardworking, responsible, and eager to integrate. On the other hand, there are also stereotypical or reserved perceptions, especially among those who have not had direct contact with members of the Romanian community. These negative perceptions are related to indirect sources (media, rumors) rather than personal experiences.

The content of the interviews confirmed the complexity of the migration phenomenon and showed that integration is not only a matter of individual will, but involves a bidirectional process, influenced by the local socio-cultural context, the openness of the host community and the dynamics of interethnic relations.

After the general analysis of the interviews with Italian citizens, a thematic structuring of the responses is useful to highlight the main dimensions that emerged from the qualitative research. The first dimension identified is related to the perception of Romanians‘ contribution to the local labor market. A significant number of participants appreciated the work done by Romanians in essential sectors such as construction, care for the elderly or cleaning services. For example, M., a 31 year old Italian who works as a construction employer, says: “Romanians are among the hardest workers I've ever had. They are not late, they do not complain, they do their job well.” These opinions reflect a majority tendency to recognize the efficiency and seriousness of Romanian workers.

A second dimension observed refers to the social integration of Romanians in Italian society. Although at the professional level they are often appreciated, their integration into local communities is perceived as partial. C., a 45 year-old teacher, remarks: “I don't really come to Italian cultural events. Maybe they should interact more, learn the language better.” This observation reflects a view shared by many respondents that the language barrier and withdrawal into small ethnic communities limit full participation in local social life.

The dimension of interpersonal interactions was also frequently present in the interviewees' answers. Those who have had direct contact with Romanians generally express positive opinions, considering them open and trustworthy. F., a 28 year old student, mentions: “I have a Romanian friend. It's open, we have a lot in common. I think people are similar, regardless of nationality.” On the other hand, people who have not had such contacts tend to take over stereotypes or opinions formed from indirect sources. This contrast suggests that direct closeness contributes to reducing prejudice.

As for the general image of the Romanian community, the opinions of the interviewees are divided. R., a 52 year old pensioner, says: “Some Romanians are ok, but I've also heard of problems with thefts… I don't know for sure how true it is.” Such remarks reflect a double perception: on the one hand, the recognition of the positive behavior of many Romanians, and on the other hand, the existence of reservations or suspicions fueled by the media. However, more than half of the respondents had a positive or neutral opinion of the Romanian community, while a significant minority expressed distrust or negative generalizations.

**Hypothesis H1:** The main reasons that determined the migration of Romanians to Florence are of an economic and family nature.

The distribution of responses regarding the reasons for emigration was analyzed by applying a Chi-square test (χ^2^), aiming to identify the existence of a significant predominance of certain motivational categories. The most frequently invoked reasons were: obtaining a better paid job (45.2%), a higher standard of living (19.4%), the desire to help the family remaining in Romania (16.1%) and family reunification (9.7%). The detailed pairwise comparisons between these categories are presented in Table 12, confirming the predominance of economic motivations.

**Table 12:** Pairwise McNemar tests comparing reasons for emigration.

**Pairwise comparison**	**McNemar χ^2^**	***p*-value (Bonferroni-adjusted)**	**Significant**
Better paid job vs. Higher standard of living	34.56	< 0.001	Yes
Better paid job vs. Family reunification	72.14	< 0.001	Yes
Better paid job vs. Helping family	41.36	< 0.001	Yes
Better paid job vs. Other reasons	72.14	< 0.001	Yes
Higher standard of living vs. Family reunification	3.12	0.076	No
Higher standard of living vs. Helping family	2.50	0.114	No
Higher standard of living vs. Other reasons	3.12	0.076	No
Family reunification vs. Helping family	0.72	0.395	No
Family reunification vs. Other reasons	0.00	1.000	No
Helping family vs. Other reasons	0.72	0.395	No

Because the five possible reasons for emigration represent mutually exclusive response options provided by the same group of respondents, the comparison of proportions requires a test suitable for paired nominal data. Therefore, a series of pairwise McNemar tests was conducted to examine whether the most frequently selected reason (“obtaining a better paid job”) differs significantly from the other categories. A Bonferroni correction was applied to control for multiple comparisons (adjusted α = 0.005).

The results show that “obtaining a better paid job” was selected significantly more frequently than all other categories of reasons for emigration. No other pairwise comparison reached statistical significance after correction. These findings indicate that economic motivations clearly predominate among Romanian migrants in Florence, supporting Hypothesis H1.

**Hypothesis H2:** The longer the period of stay in Italy, the higher the level of life satisfaction.

To verify this hypothesis, we applied the Pearson correlation test between the variable “number of years of residence in Italy” and “level of satisfaction with life” (score measured on a Likert scale from 1 to 5). The results indicated a statistically significant positive correlation: r = 0.425, *p* < 0.001, *N* = 330. It suggests that as the length of residence increases, the level of life satisfaction also tends to increase. The numerical values supporting this association can be found in Table 13.

**Table 13:** Pearson correlation between length of stay in Italy and life satisfaction.

**Variable**	***R***	***N***	***p***
Length of stay–Satisfaction	0,425	330	< 0,001

The H2 hypothesis is thus confirmed. The result is in agreement with the studies of Kogan and Shen (2019), who argue that the time spent in the host society positively influences the wellbeing felt by migrants.

**Hypothesis H3:** The Romanian community is perceived more negatively by Italian citizens than by its members.

To test H3, the perception scores of Romanians (*N* = 330) and Italians (*N* = 30) were compared. Because the variable is ordinal and the comparison involves two independent groups, a Chi-square test of independence was used instead of a *t*-test, following the reviewer's recommendation. For analysis, the 5-point scale was collapsed into three ordered categories (negative, neutral, positive), a standard procedure for ordinal variables. The Chi-square test indicated a statistically significant association between nationality and perception levels, showing that the two groups differ in how positively they evaluate the Romanian community.

These results support hypothesis H3: the Romanian community is perceived more positively by its members than by Italian citizens. Table 14 presents the descriptive statistics comparing the perceptions of Romanians and Italians. The finding is statistically significant and consistent with previous research on intergroup perception asymmetries (de Rosa et al., 2023; Blnescu and Russo, 2022).

**Table 14:** Perception of the Romanian community among Romanian and Italian respondents (descriptive statistics).

**Group**	***N***	**Mean**	***SD***
Romanian	330	4.10	0.89
Italian	30	3.20	1.02

## 4 Discussion

### 4.1 Discussions based on quantitative results

The quantitative analysis conducted among 330 Romanians residing in Florence provides coherent evidence in response to the central research question: *How do Romanians perceive the integration process and to what extent do they preserve their cultural identity while living in the diaspora?* The data confirm the validity of the hypotheses formulated and indicate that the integration of Romanians in Florence follows a pattern of functional, though selective, adaptation.

The findings reveal that the main motivations for migration are predominantly economic and family-related. This outcome aligns with the classical *push-pull* model formulated by Lee (1966), which explains migration as a balance between opportunities in the destination and pressures in the country of origin, as well as with the theory of migratory networks developed by Massey et al. (1993). Romanian scholars such as Sandu (2010) and Anghel (2009) reached similar conclusions, emphasizing that migration decisions are shaped not only by financial factors but also by the existence of informal support systems that facilitate relocation and settlement abroad. The data from the present study reinforce this dual perspective, showing that the migration of Romanians to Florence is guided both by pragmatic needs and by the relational dynamics of family and community.

Most respondents declared that they initially migrated alone, which suggests that the first wave of migration was exploratory, followed by a gradual process of consolidation through social and family networks. These findings correspond to Ban's (2012) concept of *economic transnationalism*, describing migration as a self-sustaining process in which early migrants pave the way for later family reunification. The pattern is consistent with Massey's view that migration becomes cumulative through interpersonal connections, which create both material and symbolic pathways of integration in the host society.

Statistical analyses also indicated significant associations between marital status and the frequency of return to Romania. Married respondents reported more frequent visits home, which underlines the continued importance of familial bonds and emotional connections with the country of origin. This behavior reflects what Sandu (2010) describes as cyclical migration, where movement between origin and destination countries represents a sustainable equilibrium rather than a definitive relocation.

When considering indicators related to quality of life—such as satisfaction, access to services, professional stability, and social participation—the results point to moderate to high levels of wellbeing and functional adaptation. The confirmation of the positive correlation between length of stay and life satisfaction supports previous findings by Kogan and Shen (2019) and Kogan et al. (2018), who observed that longer residence in the host country fosters both economic security and psychological stability. At the same time, the data reveal a form of *selective integration*: while Romanians achieve professional stability and social respect, full participation in cultural and civic life remains limited. This situation reflects the model of *bicultural integration* described by Berry (2005), according to which migrants manage to reconcile professional adaptation with the preservation of their cultural identity.

The comparative analysis of perceptions between Romanians and Italians confirms statistically significant differences regarding the image of the Romanian community. These divergences illustrate how mutual representations continue to be influenced by stereotypes, a finding consistent with the works of de Rosa et al. (2023), Blnescu and Russo (2022), and Tarabusi (2022). The persistence of these symbolic boundaries shows that integration depends not only on the migrants' adaptation efforts but also on the openness of the host society. Nevertheless, both the survey and the qualitative interviews highlight that direct interpersonal contact between Romanians and Italians tends to generate positive attitudes, mutual trust, and cooperation, which progressively counteract preconceptions rooted in media discourse.

Overall, the results depict a nuanced image of Romanian migration to Florence. The integration process is partial but effective, supported by economic motivation, sustained by social networks, and facilitated by gradual adaptation to the host society. At the same time, cultural identity is not lost but reshaped through bicultural negotiation. Integration thus appears as a reciprocal and evolving process, in which both migrants and members of the host community redefine their social positions through daily interaction and shared experience.

### 4.2 Discussions based on qualitative results

The qualitative component of the study, based on semi-structured interviews with 30 Italian citizens from the Florence region, complements the quantitative analysis by providing an external and interpretative perspective on the Romanian community. Through these narratives, the voices of the host population reveal how everyday interactions shape perceptions of migration, integration, and cultural coexistence. The insights gathered from these interviews contribute substantially to the understanding of the research question and deepen the contextual interpretation of the findings.

The data indicate that Italians' perceptions of Romanians' motivations for migration and their quality of life in Italy are strongly influenced by the nature and intensity of direct contact. Respondents who had positive experiences—whether in the workplace, as neighbors, or through social exchanges—tended to describe Romanians as hardworking, responsible, and well-integrated individuals. These statements confirm the quantitative conclusions regarding professional adaptation and economic participation and align with the observations of Giuliani et al. (2023) and Santini and Fabbietti (2023), who underline the positive recognition of migrants employed in sectors such as construction and care work.

Conversely, a segment of the interviewees expressed hesitation, stereotypes, or mistrust toward the Romanian community, particularly when they lacked personal contact with migrants. This polarization reflects a pattern frequently reported in migration studies, where social representations are shaped by indirect sources of information. As (Grigoraş 2017) and de Rosa et al. (2023) emphasize, mass media often play a decisive role in reinforcing prejudiced narratives and amplifying negative images of migrant groups. Similarly, Romero (2017) notes that the socio-political climate can transform migration into a perceived threat, especially in periods of economic uncertainty or public anxiety.

The interviews also highlight the transformative role of interpersonal contact in reducing prejudice and fostering mutual understanding. This finding resonates with the analyses of Uccellini (2012) and Poenaru (2020), who argue that migrants' active participation in local communities and civic life strengthens their public image and accelerates social integration. The participants who engaged in sustained collaboration with Romanian workers or neighbors tended to perceive them as trustworthy and culturally adaptable, illustrating the positive impact of everyday encounters on intercultural relations.

Taken together, the qualitative findings reinforce the broader interpretation of integration as a reciprocal and dynamic process. Integration depends simultaneously on migrants' willingness to adapt and on the inclusiveness of the host society. This dual perspective corresponds with the literature on multi-level governance in migration, which emphasizes the importance of coordinated local and national efforts to promote inclusion and dialogue. As Panizzon and Van Riemsdijk (2019) and Pettrachin (2024) argue, successful integration policies are built upon local initiatives that encourage interaction, reduce cultural distance, and support migrants' agency within the host community.

Overall, the qualitative evidence confirms that integration is not a one-sided adjustment, but a shared social construction sustained by interpersonal contact, community participation, and institutional openness. It complements the quantitative findings by situating them within the lived experiences and perceptions of the host population, offering a nuanced and multidimensional understanding of Romanian migration in contemporary Florence.

### 4.3 Correlation between quantitative and qualitative results

The integration of quantitative and qualitative data provides a nuanced and multidimensional understanding of the experience of Romanians living in Florence. The two perspectives complement and validate one another, creating a coherent interpretative framework in which the statistical trends identified through the survey are reinforced by the perceptions revealed in the interviews with Italian citizens. This mixed-method approach thus allows for a deeper exploration of migration, integration, and identity, linking the migrants' self-perceptions with those of the host community.

The quantitative results highlighted that economic and family-related motivations are the dominant reasons for migration, confirming that the decision to leave Romania is driven by pragmatic and relational factors rather than individual spontaneity. This conclusion is reinforced by the qualitative findings, where Italian respondents frequently described Romanians as hardworking, responsible, and reliable—qualities especially appreciated in construction, domestic care, and other essential sectors. Such positive assessments indirectly validate the migrants' own perception of their role within the local economy, demonstrating that professional diligence constitutes an important channel of social recognition.

When analyzing social integration and life satisfaction, the correlation between the length of stay in Italy and the perceived level of satisfaction indicates that integration occurs primarily at a functional level, with professional stability preceding cultural participation. This interpretation is consistent with the qualitative testimonies, which emphasize that although Romanians are well integrated into the labor market and are regarded as dependable workers, their involvement in community events and intercultural activities remains limited. Consequently, integration appears to be more pragmatic than relational, shaped by work-based interaction rather than deep social exchange.

The dimension of interpersonal relationships further illustrates this selective integration. Quantitative data showed that Romanians generally feel respected and accepted in Italy, and this tendency is mirrored in the qualitative accounts, where Italians who have had direct contact with Romanians expressed positive and friendly attitudes, free from stereotypes. In contrast, in cases where interaction was absent, perceptions were often influenced by negative media narratives, leading to more critical or distorted views. This asymmetry clarifies the statistically confirmed differences in perception (related to Hypothesis H3), suggesting that prejudice persists primarily in contexts lacking personal contact, whereas genuine encounters foster understanding and trust.

Viewed together, these complementary results strengthen the validity of the study through methodological triangulation. The convergence between statistical correlations and interview-based insights supports a coherent interpretation of the integration process as both partial and functional. Migration emerges as a dynamic negotiation between adaptation and identity preservation, in which economic participation ensures visibility and stability, while cultural distinctiveness remains a marker of belonging.

In conclusion, the integration of quantitative and qualitative findings confirms the value of a mixed methodological approach for exploring complex social phenomena such as migration and integration. The combined perspectives offer a comprehensive image of the Romanian diaspora in Florence—an image that highlights mutual influences, reciprocal perceptions, and the ongoing negotiation between social inclusion and cultural identity.

## 5 Limitations

Although the results obtained provide a relevant and coherent picture of the integration process and cultural identity among the Romanian community in Florence, the study has some inherent limitations.

First of all, the restricted geographical character of the research, focused on the Florence area, may limit the generalization of the conclusions to the entire Romanian diaspora in Italy. Participation in qualitative research was also voluntary, which may influence the level of openness and honesty of the answers, especially in the case of interviews with Italian citizens.

Another aspect is that the research tools, although validated, do not fully capture the complexity of identity and intercultural dimensions. In addition, being a cross-sectional study, the evolution of the integration process over time was not followed, but only the perceptions from a specific moment.

However, by combining quantitative and qualitative components and by referring to a solid theoretical basis, the research offers valuable insights into the realities of the Romanian diaspora and opens relevant directions for future research.

## 6 Conclusions

The present research aimed to explore the way in which Romanians settled in Florence perceive their integration into Italian society and to what extent they preserve their cultural identity in the context of life in the diaspora. The combination of quantitative analysis with qualitative exploration allowed to outline a nuanced and balanced picture of the realities experienced by this community.

The results confirm that the main motivations behind migration are economic and family-related, in line with the literature that has identified these reasons as dominant for post-accession Eastern European migration. The data also show that a longer duration of residence in Italy is associated with an increased level of life satisfaction, but does not guarantee full social or cultural integration. These findings support the idea of selective integration, in which migrants choose to adopt those elements of the host society that facilitate their stability, without giving up their own values and identity.

A key aspect of the study was the analysis of divergent perceptions between migrants and members of the host society. While the Romanians interviewed perceive themselves as well integrated, hardworking and respectful, some voices among Italian citizens express reservations or stereotypes. This contrast reveals the importance of authentic intercultural dialogue and direct contact in reducing prejudice, being an essential direction for social interventions and public policies.

Another major result of the research is the active maintenance of the Romanian cultural identity. It is not perceived as a barrier in the integration process, but as an important symbolic resource. Migration seems, in some cases, to reinforce the sense of belonging, not to dilute it—a relevant observation for understanding identity processes in the context of international mobility.

In light of these findings, the study contributes to the literature by offering a local perspective, but deeply connected to European trends on mobility, integration and identity in the diaspora. The mixed approach allowed not only the quantification of the phenomenon, but also the understanding of the feelings and social representations involved.

In conclusion, the research results support the validity of the formulated hypotheses and provide a pertinent answer to the research question. The integration of the Romanian community in Florence is complex, partial, but functional, and the national identity is actively maintained. These elements should be taken into account in the development of social and educational policies for migrants. The study can also be a starting point for future extensive longitudinal or comparative research investigating the phenomenon in different cultural contexts or in other regions of Italy and Europe.

## Data Availability

The raw data supporting the conclusions of this article will be made available by the authors, without undue reservation.
